# The Aromatic Amino Acid Biosynthesis Gene *VdARO2* and the Cross-Pathway Regulator *VdCPC1* Coordinately Regulate Virulence in *Verticillium dahliae*

**DOI:** 10.3390/microorganisms13122852

**Published:** 2025-12-15

**Authors:** Chongbo Zhang, Can Xu, Yuan Wang, Jiafeng Huang, Xiaoqiang Zhao

**Affiliations:** 1 Key Laboratory of Oasis Agricultural Pest Management and Plant Protection Resources Utilization, College of Agriculture, Shihezi University, Shihezi 832000, China; 16699046676@163.com (C.Z.);; 2 Xinjiang Academy of Agricultural and Reclamation Sciences, Shihezi University, Shihezi 832000, China

**Keywords:** aromatic amino acid biosynthesis, chorismate synthase, cross-pathway control, pathogenicity

## Abstract

The soil-borne fungus *Verticillium dahliae* is a devastating pathogen responsible for substantial losses in cotton production. This study elucidated the key functions of *VdARO2* and *VdCPC1* in fungal pathogenicity. *VdARO2* encodes a Chalmoic acid synthase involved in the biosynthesis of aromatic amino acids, while *VdCPC1* is a central regulator of amino acid starvation response and reveals a key regulatory relationship between *VdARO2* and *VdCPC1* to jointly control fungal virulence. We demonstrate that both genes are essential for growth, conidiation, and microsclerotia formation in *V. dahliae*. The *VdΔaro2* mutant exhibited severe developmental defects and a complete loss of microsclerotia production, accompanied by widespread transcriptional dysregulation. Disruption of *VdARO2* significantly upregulated *VdCPC1*, triggering a compensatory starvation response that nonetheless failed to restore pathogenicity. Silencing *VdCPC1* similarly impaired fungal development and attenuated virulence. Our findings reveal a crucial regulatory axis in which *VdARO2* and *VdCPC1* coordinate metabolic homeostasis and stress adaptation to facilitate host colonization, thereby identifying promising targets for the control of *Verticillium* wilt.

## 1. Introduction

Cotton, a crop of immense economic importance, is highly vulnerable to various biotic and abiotic stresses throughout its growth cycle, including temperature fluctuations, bacterial, nematode, viral, and fungal infections. These adversities lead to substantial losses in both yield and fiber quality [1,2,3]. Among them, *Verticillium* wilt, caused by the soil-borne fungus *Verticillium dahliae*, stands out as one of the most devastating diseases. After successful infection of roots, the key to the pathogenic cycle of *V. dahliae* is its strong systemic colonization ability. Mycelium or conidia first germinate in the rhizosphere and establish primary infection sites through root wounds or direct penetration of the root epidermis. Subsequently, the pathogen must break through the key barriers such as the endodermis, and finally successfully invade the vascular system of the plant–xylem vessel. Once it enters the xylem, the pathogen faces severe challenges from the host environment, including nutrient deficiency (especially amino acid scarcity), the flow of plant defense compounds, and physical limitations of the vessel structure. In order to survive and spread in this harsh environment, the pathogen has adopted a series of adaptive strategies: the mycelium grows along the vessel wall and produces a large number of conidia, which can be passively and long-distance transported with the transpiration flow, so as to realize the systematic spread from the local infection point to the whole plant. The final mass multiplication of hyphae and conidia physically blocks water transport [4,5,6], which is one of the direct causes of plant wilting symptoms. Given the limitations of current control methods, developing resistant cultivars through genetic engineering represents the most sustainable solution. However, the scarcity of resistance genes in available cotton germplasms severely hinders such efforts. Therefore, a deep understanding of the molecular interactions between *V. dahliae* and its host is essential to devise novel control strategies.

Amino acids are central to fungal pathogenicity, serving not only as fundamental nitrogen and carbon sources for growth and proliferation but also as critical regulators of virulence. For instance, the plant pathogenic fungus *Magnaporthe oryzae* relies on its amino acid permease *MoGap1* (belonging to the amino acid transporter family) to sense and absorb amino acids, thereby regulating the TOR signaling pathway and autophagy process. Deletion of *MoGap1* leads to a decrease in spore production, an increase in cell wall stress sensitivity, and a significant decrease in virulence [7]. In the process of infecting plants, *V. dahliae* often faces the environmental pressure of lack of nitrogen source in the host vascular bundle. At this time, the fungus will activate its own amino acid synthesis pathway to meet the growth needs. The *VlCPC1* of *Verticillium longisporum* is involved in the regulation of amino acid synthesis, and its deletion will significantly inhibit the growth of fungi in an amino acid-deficient environment and reduce pathogenicity. Beyond biosynthesis substrates, amino acids and their metabolites directly modulate pathogenic processes such as hyphal morphogenesis and toxin production. In *C. albicans*, proline degradation activates the cAMP/PKA signaling pathway, inducing the transition from yeast to invasive hyphae—a key mechanism for tissue penetration [8]. Similarly, *Fusarium oxysporum* in glycine influences chlamydospore formation, and alterations in amino acid synthesis pathways correlate with toxin production and pathogenicity [9]. The growth and spore formation of *V. dahliae* are highly dependent on external amino acids as nitrogen sources. There were significant differences in the promoting effects of different amino acids on mycelial growth and microsclerotia formation, while the absence of the aromatic amino acids tryptophan and phenylalanine significantly delayed the growth rate [10]. Fungi also exploit amino acid metabolism to remodel the host microenvironment, thereby enhancing survival and promoting immune evasion. For example, nitrogen is a key element for the synthesis of amino acids and nucleic acids by pathogenic fungi, and directly affects the infection strategy of pathogens and the defense mechanism of host plants [11]. ammonia released during amino acid catabolism can neutralize the acidic phagosome environment, facilitating yeast-to-hypha transition and immune escape, a process dependent on regulators like *Stp2p* [12,13]. In *Colletotrichum higginsianum*, the deletion of arginine biosynthetic gene *arg* significantly reduces the pathogenicity of the pathogen to *Arabidopsis thaliana*, indicating that arginine metabolism is the key to pathogenicity [14]. The lack of amino acid transporter LHT1 in *A. thaliana* leads to the lack of glutamine, which enhances broad-spectrum disease resistance through the salicylic acid pathway, indicating that the homeostasis of specific amino acid glutamine regulates the host immune response [15]. Thus, amino acids function not only as essential nutrients but also as key pathogenic signals and effectors.

Fungi are prototrophic for amino acids, and their biosynthesis is crucial for survival, especially under nutrient limitation [16]. The shikimate pathway, absent in mammals, is a key route for synthesizing aromatic amino acids. Chorismate synthase, which catalyzes the conversion of 5-enolpyruvylshikimate-3-phosphate (EPSP) to chorismate, is essential in this pathway [17,18,19,20]. Chorismate serves as the precursor for tryptophan, phenylalanine, and tyrosine, and defects in its synthesis—as seen in yeast *Δaro2* mutants—lead to severe growth impairment, reversible upon aromatic amino acid supplementation [21]. In *Verticillium longisporum*, silencing *Vlaro2* results in non-viability under amino acid starvation and attenuated virulence [22], highlighting the enzyme’s importance.

In fungi, the process of amino acid uptake is highly complex. When environmental amino acids are abundant, fungi preferentially scavenge these extracellular nutrients, thereby reducing de novo biosynthesis to conserve cellular energy [23]. Conversely, under amino acid deprivation, fungi rapidly activate their biosynthetic machinery by upregulating the transcription of relevant genes [24]. This adaptive response is often mediated by the cross-pathway control (CPC) system, a conserved mechanism that rectifies amino acid imbalance across various fungi [25]. Key regulators of this system include Gcn4 in *Saccharomyces cerevisiae* [26], *CPC1* in *Neurospora crassa* [27], and *CPCA* in *Aspergillus niger* [28]. These regulators are activated under amino acid starvation. In *S. cerevisiae*, for instance, the protein level of Gcn4 is tightly controlled: under sufficiency, it is rapidly degraded via ubiquitin-mediated proteolysis, whereas during starvation, its translation is enhanced and its degradation is suppressed, leading to rapid accumulation [29,30,31,32]. Beyond amino acid regulation, the CPC regulator (Gcn4/CpcA/Cpc1) also plays a vital role in fungal growth and development [33,34]. This function is particularly critical for pathogens like *V. longisporum*, which must cope with the amino acid-scarce environment of the host xylem. Silencing of *VlCPC1* in this pathogen results in heightened sensitivity to amino acid starvation, impaired development, and attenuated virulence [35].

In *V. longisporum*, silencing the *Vlaro2* gene upregulates the cross-pathway regulatory gene *VlCPCA*. This upregulation is observed not only in the mutant but also in the wild-type fungus during plant colonization, suggesting that inducing the cross-pathway control is necessary for the fungus to overcome amino acid imbalances within the xylem. Recent studies have found that the biosynthesis of branched-chain amino acids in *V. dahliae* is the key to its growth and pathogenicity. More importantly, when the pathway is interrupted by gene knockout, the pathogen significantly up-regulates the expression of the cross-pathway control (CPC) core transcription factor *VdCPC1*. This finding strongly suggests that the CPC system plays a central role in the response of *V. dahliae* to amino acid stress in vivo [36]. However, after *VdCPC1* is activated, it is unclear which target genes are specifically regulated downstream to reshape the amino acid metabolic network to help the pathogen adapt to the host environment. Based on studies in other species of the genus Verticillium (e.g., *V. longisporum*), the activation of the CPC system is often accompanied by an up-regulation of the aromatic amino acid synthesis pathway to compensate for the lack of specific amino acids [37]. The first key branch point of aromatic amino acid synthesis is chorismate, which is catalyzed by chorismate synthase (encoded by *ARO2* gene). In *V. dahliae*, we hypothesized that *VdCPC1* may coordinate the homeostasis of amino acid metabolism by regulating the expression of *VdARO2*, a key gene for aromatic amino acid synthesis, thereby affecting its pathogenicity. However, whether there is a direct regulatory interaction between these two key nodes, one responsible for core metabolism and one responsible for global regulation and how this interaction accurately affects the host adaptability of pathogens remain unknown. In order to verify this hypothesis and further study the relationship between *VdARO2* and *VdCPC1* in *V. dahliae*, we used host-induced gene silencing (HIGS) and artificial small interfering RNA (asiRNA) techniques to study the function of *VdCPC1*. At the same time, we characterized the *VdARO2* encoding isocitrate synthase by targeted gene deletion and transcriptome analysis to elucidate its role in pathogenicity. This study provides a basic understanding of the molecular mechanism of *V. dahliae* virulence.

## 2. Materials and Methods

### 2.1. Test Strains and Culture Conditions

The virulent defoliating *V. dahliae* V592 isolated from cotton in Xinjiang was used as the wild-type strain in this study [38,39]. The gene knockout strains were generated from V592 strain. The plasmid *pGKO-HPT* carried hygromycin B resistance gene and kanamycin resistance for gene knockout, and plasmids *pTRV2* and *pTRV1* for gene silencing. *Escherichia coli* DH5α and *Agrobacterium tumefaciens* EHA105 were preserved in our laboratory for plasmid propagation and fungal transformation. All the fungal strains were cultured on potato dextrose agar (PDA: 200 g potato, 15 g glucose, 15 g agar powder) plates at 25 °C for phenotypic characteristics, DNA and RNA extraction, cultured in liquid Czapek-Dox medium (CDM: 2 g NaNO_3_, 1 g K_2_HPO_4_, 0.5 g KCl, 0.24 g MgSO_4_, 0.01 g FeSO_4_·7H_2_O) at 25 °C with shaking at 200 rpm/min to collect conidia for infection experiments. The plasmids used in this study were obtained as follows: the gene knockout vector *pGKO-HPT* was kindly provided by Professor Huishan Guo from the Institute of Microbiology, Beijing, China. The vectors *pTRV1*, *pTRV2* (for host-induced gene silencing) [40], and *p1300-Neo-oLiC-Cas9-TtrpC* [41] (for genetic complementation) were maintained in our laboratory. All plasmids were propagated and preserved by transforming into *E. coli* DH5α competent cells via the heat-shock method.

### 2.2. Sequence Analysis of VdARO2 and VdCPC1 Genes in V. dahliae

The wild-type strain V592 was cultured on PDA medium. After one week, the fungal tissue was scraped and the DNA was extracted according to the instructions of the Biospin fungal genomic DNA extraction kit (BIOER, Hangzhou, China). The V592 strain was inoculated into Czapek-DOX medium for shaking culture 5 days, and then the conidia were collected. RNA was extracted from the collected conidia according to the Trizol method (ThermoFisher, Waltham, MA, USA), and cDNA was synthesized with Synthesis SuperMix kit (TaKaRa, Dalian, China). Primers VdARO2full-F/R and *VdCPC1*full-F/R were designed according to the complete sequences of *VdARO2* (VDAG_07695) and *VdCPC1* (VDAG_10113) genes in NCBI database (https://www.ncbi.nlm.nih.gov/ (accessed on 2 May 2024)). The full-length of *VdARO2* and *VdCPC1* genes were amplified using the DNA and cDNA of the wild-type strain V592 as templates. The PCR products were purified and sent to Company for sequencing. The primers used are shown in Supplementary Table S1.

The protein sequences of VdARO2 and VdCPC1 were analyzed by MEME program (https://meme-suite.org/meme/tools/meme (accessed on 15 March 2024)). The amino acid sequences of VdARO2 and VdCPC1 homologous proteins were obtained by BLASTp search in NCBI (Protein BLAST: search protein databases using a protein query (https://blast.ncbi.nlm.nih.gov/Blast.cgi?PROGRAM=blastp&PAGE_TYPE=BlastSearch&LINK_LOC=blasthome, accessed on 20 March 2024)) with the full-length amino acid sequences of VdARO2 and VdCPC1. Phylogenetic tree analysis was performed using software MEGA 7.0 and the bootstrap test was replicated 1000 times.

### 2.3. Knockout and Complementation of VdARO2 Gene in V. dahliae

The *VdARO2* gene was replaced by the hygromycin B resistance gene (*HPH*) based on the principle of homologous recombination. The upstream (1143 bp) and downstream (1048 bp) homologous fragments of the *VdARO2* gene were amplified by primers *VdARO2*-up-F/R and *VdARO2*-down-F/R using the genomic DNA of V592 as a template (Supplementary Table S1). The purified PCR product was recombined with the linearized *pGKO-HPT* vector by ClonExpress MultiS One Step Cloning Kit (Vazyme, Nanjing, China). The vector was then transformed into the V592 strain by *A. tumefaciens*-mediated transformation method (ATMT) to obtain the knockout mutant strain [42]. To genetic complementation, the full-length *VdARO2* genomic sequence (1308 bp) was amplified from the genomic DNA of wild-type V592 by Vd*Δaro2/VdΔAro2*-F/R primers. The full length of the gene was ligated into the linearized *p1300-Neo-oLiC-Cas9-TtrpC* vector digested with *Xba*I and *BamH*I [41]. The vector was transformed into the knockout mutant of the gene by the ATMT method to obtain a complementary mutant strain.

### 2.4. Determination of Phenotypic Characteristics of Fungi

A total of 2 μL of 1.0 × 10 CFU/mL conidial suspension was inoculated into PDA medium and cultured at 26 °C, with wild-type strain V592 as the control. The colony diameter of each strain was measured and the average growth rate of the colony was calculated on the 5 and 15 days post-inoculation (dpi). Each strain was set up 3 replicates.

Conidia suspension (1 mL, 1.0 × 10^6^ CFU/mL) was added to 100 mL Czapek-Dox medium and incubated at 26 °C, 200 rpm/min for 7 days. The number of conidia was measured every 24 h. Each strain was set up 3 replicates.

Conidial suspension with a concentration of 1.0 × 10^7^ CFU/mL was coated on Basal minimal medium (BMM) covered with cellophane, and the formation of microsclerotia was observed under a microscope after 14 days. In addition, the microsclerotia on the cellophane were scraped for wet weight measurement. Each strain was set up 3 replicates.

### 2.5. Determination of Pathogenicity

The susceptible cotton variety “Junmian No.1” was selected for the determination of pathogenicity. When the cotton grew to fifth true leaf stage, the spore suspension with a concentration of 1.0 × 10^7^ CFU/mL was inoculated by unimpaired root-dip inoculation method and the infection was soaked for 40 min. Each strain was inoculated with three pots and twelve cotton seedlings per pot. Specific methods as previously described [43]. The disease index was calculated as described by Zhang et al. [44]. After 30 dpi, the stems of cotton were collected. In order to observe the colonization of internal vascular bundles, a cross-sectional observation was carried out at the first stem node of cotton with a sterile blade. The exposed vascular tissue was then immediately examined under a stereoscope (Olympus MVX10, Tokyo, Japan) for discoloration (browning), which serves as a key indicator of disease severity [45].

### 2.6. Host-Induced Silencing of VdCPC1 Gene

The fragment of *VdCPC1* gene (666 bp) was amplified by primer *VdCPC1*-HIGS-F/R using V592 cDNA as template. The *pTRV2* vector was digested with restriction endonucleases BamHI and KpnI. Subsequently, the linearized vector *pTRV2* was ligated with the PCR purified product by ClonExpress II One Step Cloning Kit (Vazyme, Nanjing, China), and the recombinant vector *pTRV2-VdCPC1* was obtained by electroporation into *A. tumefaciens* strain EHA105. The negative control empty vector *pTRV2:00* and the positive control *pTRV2:GhCHLI* were also transformed into the same *A. tumefaciens* strain (EHA105) by electroporation, respectively. Healthy cotton seedlings with fully expanded cotyledons growing for about 10 days were selected. A 1 mL sterile syringe (remove the needle) was used to suck the prepared Agrobacterium suspension. The seedlings were gently fixed with one hand, and the front end of the syringe was tightly attached to the back of the cotyledon (abaxial surface) with the other hand. In order to ensure the effective penetration of the bacterial solution into the tissue, we chose to slowly inject at multiple sites of the cotyledon (usually 2–3 points per cotyledon), and apply a gentle and stable pressure at the same time until the bacterial solution formed a water-immersed plaque around the contact point [46]. The inoculated plants were cultured under normal conditions. *pTRV2-GhCHLI*, which targets a cotton chlorophyll biosynthesis gene (Mg-chelatase I subunit), was included as a positive control for VIGS efficiency [47]. The appearance of leaf albinism in these plants two weeks post-inoculation confirmed the successful establishment of the gene silencing system throughout the plant, thereby validating the experimental timeline and setup for subsequent pathogenicity assays. Two weeks later, when the leaves of *pTRV2-GhCHLI* positive control plants appeared albino, the silencing efficiency of *VdCPC1* in cotton leaves was confirmed by qRT-PCR and the cotton plants treated with *pTRV2-VdCPC1* and *pTRV2:00* were inoculated by root irrigation [48,49]. Each cotton was inoculated with 20 mL of V592 spore suspension at a concentration of 1 × 10^7^ CFU/mL, and the incidence of cotton was observed at 14 and 21 dpi. The investigation of disease severity and the calculation of disease index were performed as mentioned above.

### 2.7. asiRNA Design and Treatment of VdCPC1 Gene

Two asiRNA sequences of *VdCPC1* gene were designed by online software (http://biodev.extra.cea.fr/DSIR/DSIR.html (accessed on 2 May 2024), https://www.invivogen.com/sirnawizard/design.php (accessed on 2 May 2024)). The use of two non-overlapping asiRNAs (asiR787 and asiR1014, corresponding to their respective target nucleotide positions) served to confirm that the observed phenotypic effects were due to specific silencing of *VdCPC1* and not due to off-target effects. In order to prevent off-target, blastn was used to detect the specificity of two asiRNA sequences to the genome sequence of *V. dahliae*. The asiRNA sequence was sent to General biol (Anhui, China) for synthesis, in which the specific sequence of the non-target organism genome was used as a negative control. The asiRNA sequences shown in Supplementary Table S1. The wild-type V592 strain was mixed with 200 nM asiRNA, and the culture conditions were 26 °C, 200 rpm. After a week, the conidia were collected and the concentration was adjusted to 1 × 10^7^ CFU/mL [40]. The phenotypic characteristics of fungi were determined according to the method described in Section 2.3. Among them, wild-type V592 was the control (CK), and V592 co-cultured with nematode asiRNA was the negative control (NC).

The susceptible cotton variety “Junmian No.1” was used as the test material. When the cotton grew to the two-leaf stage, the infection experiment was carried out by the spore suspension root irrigation method. V592 and 200 nM asiRNA spore suspension were adjusted to 1 × 10^7^ CFU/mL for inoculation, and each cotton was inoculated with 20 mL. The incidence of cotton was observed at 14 and 21 dpi. There were 20 cotton plants in each treatment, and the disease index of cotton seedlings was calculated according to the above. The wild-type V592 was the control (CK), and V592 co-cultured with nematode asiRNA was the negative control (NC).

### 2.8. Plate Assay of 5-Methyl-Tryptophan

All strains were inoculated on Czapek-Dox Agar (CDA) medium with or without 5 mM 5-methyl-tryptophan (5-MT) and cultured at 26 °C. The analog induces a false-feedback inhibitor, trypthophan starvation. The colony diameter of each strain was measured and the average growth rate of the colony was calculated on the 5 and 15 dpi. Each strain was set up 3 replicates. All strains were inoculated into Czapek-DOX medium, and then 5 mM 5-MT was added for induction. The expression of target genes was detected by RT-qPCR.

### 2.9. Gene Expression Analysis

RNA was extracted and cDNA was synthesized according to the above method, cotton leaves were collected and RNA was extracted according to EASYspinPlus Plant RNA Extraction Kit (Aidlab, Beijing, China). Quantitative real-time PCR reaction was performed using the SYBR Green Mix (TaKaRa, Dalian, China) on the 7500 real-time PCR system. The program is 95 °C for 10 s, 40 cycles at 60 °C for 15 s, and 72 °C for 20 s. The fungi *β-tubulin* (*DQ266153*) gene and the cotton *GhUBQ7* gene were used as internal reference genes and the results were analyzed by the 2^−ΔΔCT^ method. The reference genes, fungal *β-tubulin* and cotton *GhUBQ7*, were selected for their stable expression under our experimental conditions [50,51].

To ensure the specificity and reliability of qPCR data, we implemented a stringent quality control procedure. Each sample was run with at least five biological replicates. Amplification specificity was verified by analyzing melting curves for each reaction. Replicates exhibiting atypical melting profiles were considered technical outliers and excluded from further analysis. Only replicates with a single, well-defined melting peak were used for calculating relative gene expression. This conservative approach ensured that the reported expression differences reflect true biological variation rather than technical artifacts. Representative melt curves are provided in Figure S3. The primers used are shown in Supplementary Table S1.

### 2.10. Transcriptomic Analysis

The wild-type V592 and the knockout mutant strain *VdΔaro2* were inoculated into the Czapek-Dox medium, and the same amount of sterile cotton roots were added. The strains were collected at 24 h and quickly frozen in liquid nitrogen. RNA was extracted from the samples according to the above method and RNA concentration and integrity were detected by Nanodrop2000 spectrophotometer (Thermo Scientific, Waltham, MA, USA) and Agient2100 biological analyzer. After passing the test, sequencing was performed by using Illumina Novaseq6000 (Beijing Biomarker Biotechnology Co., Ltd., Beijing, China). The original data was filtered to obtain clean reads, and then the clean reads were compared with the *V. dahliae* reference genome (https://www.ncbi.nlm.nih.gov/datasets/genome/?taxon=27337 (accessed on 9 Octember 2024)) to obtain mapped data. By using DESeq2, two samples (three biological replicates) were compared to obtain differentially expressed genes. In the screening of differentially expressed genes, Fold Change ≥2 and FDR < 0.01 were used as the criteria.

### 2.11. Statistical Analysis

In this study, the statistical analysis was conducted with the software of IBM SPSS Statistics 26.0. Data are presented as mean ± SD of three independent replicates. Differences between groups were analyzed by two-tailed Student’s *t*-test (* *p* < 0.05; ** *p* < 0.01; *** *p* < 0.001).

## 3. Results

### 3.1. Molecular Cloning and Phylogenetic Analysis Identified the Conservation of VdARO2 and VdCPC1 in V. dahliae

**Figure 1:**
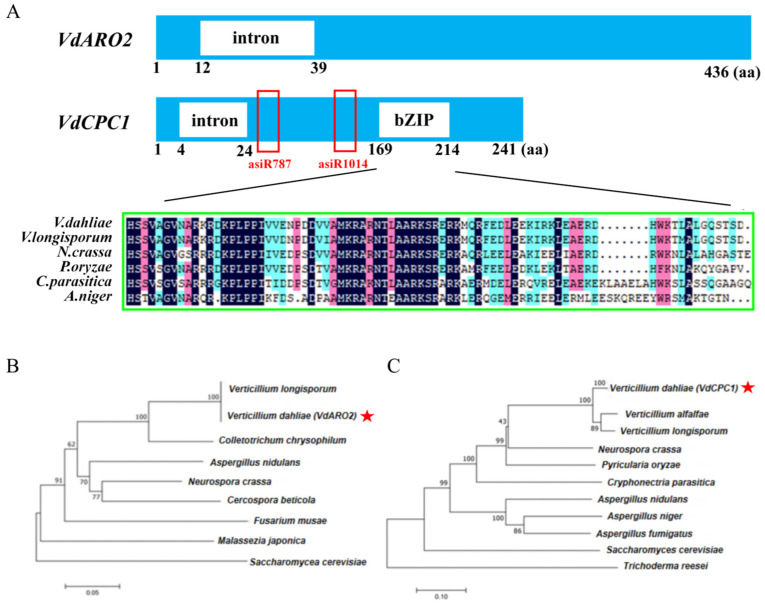
Molecular characterization and phylogenetic analysis of *VdARO2* and *VdCPC1* in *Verticillium dahliae*. (**A**) Schematic representation of the domain architecture of VdARO2 and VdCPC1 proteins. VdCPC1 is shown with a conserved bZIP domain indicated. The red box below VdCPC1 denotes the region targeted by asiRNAs. (**B**) Phylogenetic tree constructed from the amino acid sequence of VdARO2 and its homologs from other fungal species. Bootstrap values from 1000 replicates are indicated at the branch nodes. The red asterisk indicates the location of the target protein VdARO2. (**C**) Phylogenetic tree constructed from the amino acid sequence of VdARO2 and its homologs from other fungal species. Bootstrap values from 1000 replicates are indicated at the branch nodes. The red asterisk indicates the location of the target protein VdCPC1.

Recent research suggests that the CPC system plays a central role in the response of *V. dahliae* to amino acid stress in vivo and found that *VdARO2* and *VdCPC1* are up-regulated in *VdILVs* knockout mutants [23]. In order to explore the role of these two genes in regulating amino acid biosynthesis, to ensure the accuracy of the gene targets for subsequent functional analysis, we first cloned and sequenced the *VdARO2* and *VdCPC1* genes from the wild-type strain V592. The cloned sequences of *VdARO2* (*VDAG_07695*) and *VdCPC1* (*VDAG_10113*) were completely identical to the reference sequences in the NCBI database. Notably, *VdCPC1* C-terminal region contains a conserved bZIP domain (Figure 1A), which is critical for dimerization and DNA binding in filamentous fungi [52,53,54].

To explore the evolutionary relationships of VdARO2 and VdCPC1, we constructed a phylogenetic tree using their homologous amino acid sequences. Both proteins specifically clustered with their respective homologs from other *Verticillium* species, indicating that they are highly conserved across fungi (Figure 1B).

### 3.2. VdARO2 and VdCPC1 Genes Affects the Vegetative Growth and Conidiation of V. dahliae

**Figure 2:**
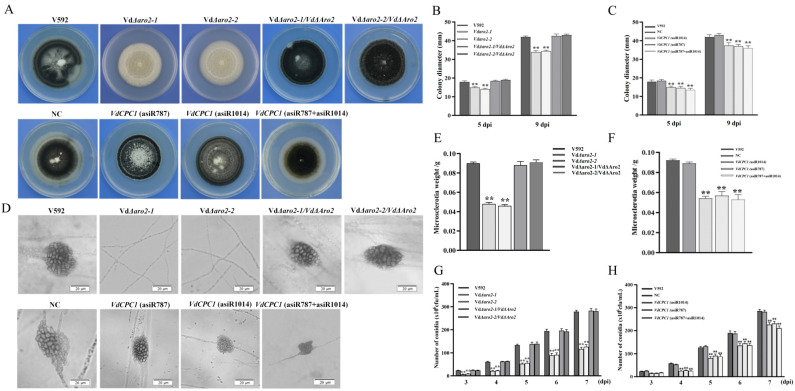
Functional characterization of *VdARO2* knockout, complementary mutants and *VdCPC1* asiRNA-treated mutants strains. (**A**) Colony morphology of wild-type (V592), *VdΔaro2* mutants, complementary mutants, *VdCPC1* asiRNA-treated strains and asiRNAs targeting nematode (NC) on PDA plates at 15 days post-inoculation (dpi). (**B**) The colony growth rates of wild-type (V592) *VdΔaro2* mutants and complementary mutants strains on PDA plates. (**C**) The colony growth rates of wild-type (V592) and *VdCPC1* asiRNA-treated strains on PDA plates. (**D**) The microsclerotia morphology of wild-type (V592), *VdΔaro2* mutants complementary mutants, *VdCPC1* asiRNA-treated strains and asiRNAs targeting nematode (NC) on BMM plate 14 days post-inoculation (dpi). (**E**) The wet weight of microsclerotia of wild type (V592) *VdΔaro2* mutants and complementary mutants strains (**F**) The wet weight of microsclerotia of wild type (V592) and *VdCPC1* asiRNA-treated strains. (**G**) Wild-type (V592), *VdΔaro2* mutants and complementary mutants strains determination of sporulation in Czapek culture medium cultured for 3–7 days. (**H**) Wild-type (V592), *VdCPC1* asiRNA-treated strains determination of sporulation in Czapek culture medium cultured for 3–7 days. Asterisks indicate significant reduction versus V592 (one-way ANOVA with Tukey’s HSD test).

To investigate the roles of *VdARO2* and *VdCPC1* in the growth and development of *V. dahliae*, the knockout vector for *VdARO2* gene and complementary mutants was constructed by using the principle of homologous recombination. The targeted gene in the genome of V592 strain was replaced with a hygromycin B resistance cassette (hygromycin B phosphotransferase gene, *HPH*) (Supplementary Figure S1A–C). All strains were cultured on PDA plates. Compared to the wild-type strain V592, the *VdΔaro2* mutants exhibited significant alterations in colony morphology. These mutants developed abundant white aerial hyphae, failed to produce microsclerotia, and showed a markedly reduced growth rate. The colony phenotypes of the *VdCPC1* (asiR787) and *VdCPC1* (asiR1014) strains were indistinguishable from the control, although their growth rates were significantly reduced (Figure 2A–C). To further investigate the roles of *VdARO2* and *VdCPC1* in microsclerotia formation, we examined all strains after 15 days of culture. Microscopic observation revealed that the *VdΔaro2* mutant failed to produce any microsclerotia. In contrast, the *VdCPC1* (asiR787) and *VdCPC1* (asiR1014) strains retained this ability, but their microsclerotia production was significantly impaired, with yields reduced by 1.7-fold and 1.6-fold, respectively, compared to the control (Figure 2D–F). These results demonstrate that both *VdARO2* and *VdCPC1* are involved in regulating radial growth and microsclerotium production in *V. dahliae*. The sporulation capacity of all strains was quantified and compared to the wild-type V592. The results demonstrated a significant reduction in conidiation for both the *VdΔaro2* mutants and the *VdCPC1* knockdown strains. At 7 dpi, the conidial production of the *VdΔaro2* mutants was reduced by 2.3-fold and 2.2-fold, respectively, while that of *VdCPC1* (asiR787) and *VdCPC1* (asiR1014) decreased by 1.2-fold (Figure 2G,H). These findings indicate that *VdARO2* and *VdCPC1* are critical regulators of conidiogenesis in *V. dahliae*.

### 3.3. Amino Acid Auxotrophy and Activation of the Cross-Pathway Control Underlie 5-MT Hypersensitivity

**Figure 3:**
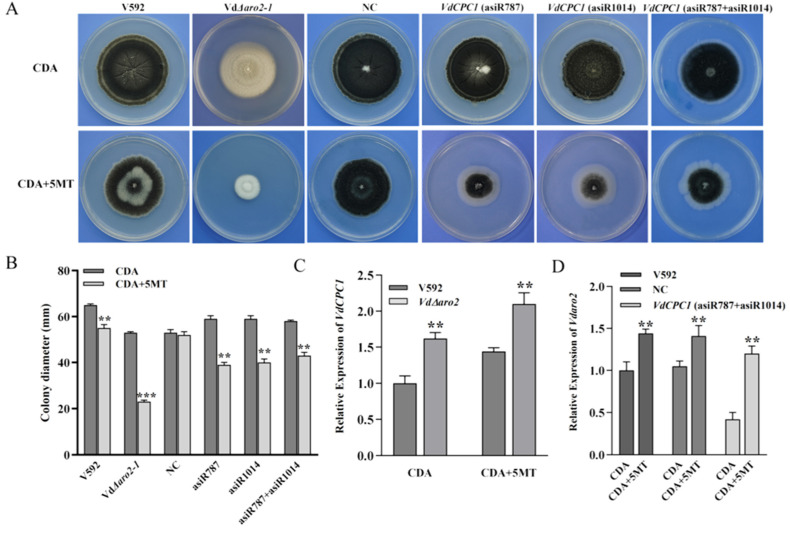
Response of *V. dahliae* strains to amino acid starvation induced by 5-methyltryptophan (5-MT). (**A**) Colony phenotypes of wild-type (V592), *VdARO2* knockout mutants (*VdΔaro2-1*, *VdΔaro2-2*), complemented strains (VdΔaro2-1/VdARO2, *VdΔaro2-2/VdARO2*), and *VdCPC1*-silenced strains grown on CDA medium with or without 5 mM 5-MT. (**B**) Quantitative analysis of colony growth rates under 5-MT stress. (**C**,**D**) Relative expression levels of VdCPC1 and VdARO2 under 5-MT treatment, as determined by RT-qPCR. Asterisks indicate a significant difference compared to the V592 strain under the same condition (one-way ANOVA with Tukey’s HSD test).

To investigate the role of *VdARO2* and *VdCPC1* in the amino acid starvation response, we challenged the strains with 5-methyl-tryptophan (5-MT), a toxic analog that induces tryptophan starvation by acting as a false-feedback inhibitor. As expected, all mutant strains, *VdΔaro2*, *VdCPC1* (asiR787), *VdCPC1* (asiR1014), and *VdCPC1* (asiR787 + asiR1014), exhibited a reduced growth rate compared to the wild-type V592 on standard medium. This growth defect was severely exacerbated when cultured on medium supplemented with 5-MT, indicating a heightened sensitivity to tryptophan starvation (Figure 3A,B).

To determine how 5-MT treatment affects the expression of *VdARO2* and *VdCPC1*, we performed RT-qPCR analysis on liquid cultures. We concurrently assessed *VdARO2* expression in the *VdCPC1*-silenced strains and *VdCPC1* expression in the *VdΔaro2* mutant. 5-MT induction significantly up-regulated *VdCPC1* expression (Figure 3C). Notably, *VdARO2* expression was also up-regulated in the *VdCPC1*-silenced background under this condition (Figure 3D). These findings suggest a potential compensatory interplay and demonstrate that both genes are essential for *V. dahliae*’s response to amino acid starvation.

### 3.4. Disruption of VdARO2 and Silencing of VdCPC1 Significantly Attenuates Fungal Virulence in Cotton

**Figure 4:**
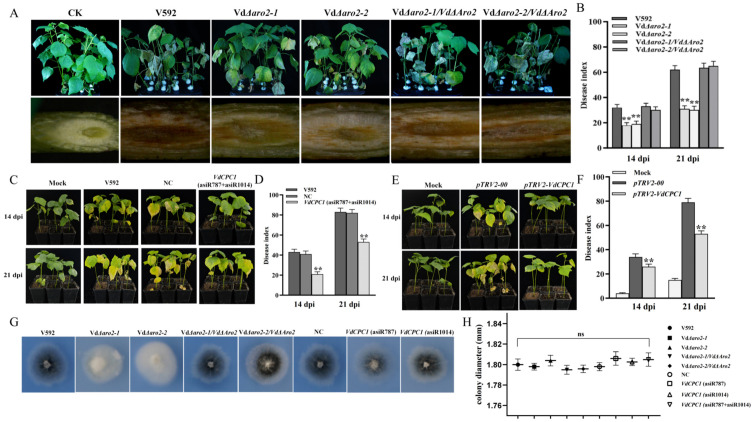
Pathogenicity assays of *VdARO2* and *VdCPC1* mutants in *V. dahliae*. (**A**) Disease symptoms and vascular browning in cotton stems 28 dpi with *VdARO2* knockout mutants. (**B**) Corresponding disease index for the assays in (**A**). (**C**) Disease symptoms on cotton plants treated with the *VdCPC1* double-silenced strain (asiR787 + asiR1014) at 14 and 21 dpi. (**D**) Disease index for the assays in (**C**). (**E**) Disease symptoms on HIGS-silenced (*pTRV2-VdCPC1*) cotton plants at 14 and 21 dpi. (**F**) Disease index for the assays in (**E**). (**G**) Hyphal penetration ability assessed on cellophane-covered minimal medium. (**H**) Colony growth rates of strains after cellophane penetration. Asterisks indicate a significant difference compared to the wild-type V592 strain (one-way ANOVA with Tukey’s HSD test).

To assess the role of *VdARO2* in the pathogenicity of *V. dahliae*, we performed cotton infection assays with the wild-type strain V592 as a control. Wilting symptoms first appeared at 8 dpi. As the disease progressed, the *VdΔaro2* mutants caused significantly milder symptoms compared to V592. By 28 dpi, the disease index for V592 reached 86.9, whereas the indices for the *VdΔaro2-1* and *VdΔaro2-2* mutants were only 50.0 and 53.1, respectively. Pathogenicity was substantially restored in the complementary strains *VdΔaro2-1/VdARO2* and *VdΔaro2-2/VdARO2*, with disease indices of 85.9 and 86.1. The restored phenotypes in these multiple independent complementation strains, coupled with PCR confirmation of *VdARO2* integration and expression (Figure S1C,D), unequivocally link the observed defects to the loss of *VdARO2* function. Furthermore, oblique sections of cotton stems at 30 dpi revealed that the vascular browning caused by V592 was the most severe, presenting as dark brown, while the mutants induced only slight discoloration (Figure 4A,B). The severity of disease symptoms was consistent with the degree of vascular browning, collectively demonstrating that *VdARO2* is essential for the full pathogenicity of *V. dahliae*.

To determine the contribution of *VdCPC1* to the pathogenicity of *V. dahliae*, we compared the virulence of *VdCPC1* knockdown strains—*VdCPC1*(asiR787) and *VdCPC1*(asiR1014) (Supplementary Figure S1D)—with relevant controls. Cotton seedlings were inoculated with wild-type V592 (CK), a nonspecific nematode asiRNA-treated V592 (NC), and the two *VdCPC1* knockdown strains. The results demonstrated a significant reduction in virulence for both knockdown strains. At 14 and 21 dpi, their disease indices (DIs of 21.3 and 53.1, respectively) were markedly lower than those of the CK (DIs of 43.2 and 83.1) and NC (DIs of 41.0 and 82.2) groups (Figure 4C,D).

To further delineate the role of *VdCPC1* in pathogenicity, we employed TRV-based host-induced gene silencing (HIGS) to target *VdCPC1* expression in *V. dahliae* during cotton infection. Once albinism appeared in the *pTRV2-GhCHLI* control plants, we confirmed the silencing efficiency of *VdCPC1* in cotton leaves. The expression of *VdCPC1* was significantly lower in *pTRV2-VdCPC1* plants than in the *pTRV2-00* controls, indicating successful gene silencing (Supplementary Figure S1E). Subsequently, these pre-silenced plants at the two-leaf stage were inoculated with *V. dahliae*. The *pTRV2-VdCPC1* plants exhibited significantly attenuated disease symptoms, with disease indices of 26.2 and 53.1 at 14 and 21 dpi, respectively, compared to 34.3 and 79.0 in the *pTRV2-00* control plants (Figure 4E,F). These results conclusively demonstrate that *VdCPC1* is essential for the full virulence of *V. dahliae*.

To determine whether the observed reduction in pathogenicity was linked to impaired hyphal penetration ability, we cultured all strains on minimal medium (MM) overlaid with cellophane. After 3 days of incubation, the cellophane was removed. All strains, including the mutants, were capable of penetrating the membrane and growing normally on the agar surface beneath (Figure 4G). Furthermore, quantification of fungal biomass before and after removal of the cellophane showed no significant difference between the mutants and the wild type (Figure 4H). These results indicate that the attenuated virulence of the *VdΔaro2* and *VdCPC1*-silenced strains is not due to a defect in their physical ability to penetrate a barrier, but rather implicates other functions of *VdARO2* and *VdCPC1* in the pathogenic process of *V. dahliae*.

### 3.5. Transcriptomic Profiling Reveals Severe Disruption of Amino Acid and Central Carbon Metabolism in VdΔaro2

**Figure 5:**
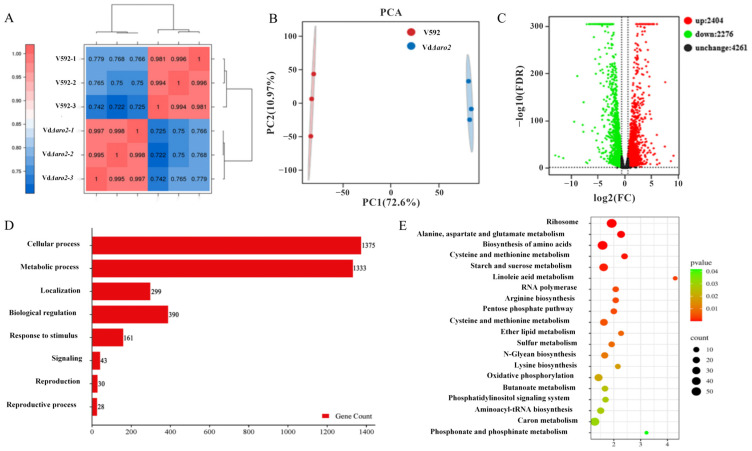
Transcriptomic profiling of the *VdΔaro2* mutant in *V. dahliae*. (**A**) Pearson correlation heatmap of biological replicates. (**B**) Principal component analysis (PCA) of transcriptome profiles. (**C**) Volcano plot of differentially expressed genes (DEGs) (threshold: |log_2_FC| ≥ 1.5 and FDR < 0.05). Red, blue, and gray dots represent significantly up-regulated, down-regulated, and non-significant genes, respectively. (**D**) Gene Ontology (GO) functional classification of DEGs. (**E**) Kyoto Encyclopedia of Genes and Genomes (KEGG) pathway enrichment analysis of DEGs. Count represents the number of enrichments.

To elucidate the transcriptional basis for the phenotypic differences between the wild-type V592 and the *VdΔaro2* mutant, we performed RNA-seq analysis. Based on the finding that *VdARO2* expression was highest at 24 h post root induction (Supplementary Figure S2), we prepared RNA from both strains at this key time point. Six libraries (three biological replicates per strain) were sequenced, yielding 44.72 Gb of Clean Data. The data quality was high, with each sample containing ~6.91 Gb of data, a Q30 score ≥98.71%, and a genome mapping efficiency of 88.92–92.36% (Supplementary Table S2). The strong correlations between replicates, as evidenced by Pearson correlation coefficients and principal component analysis, confirmed the robustness of the data (Figure 5A,B). Analysis of the transcriptome data identified 4680 differentially expressed genes (DEGs) between the wild-type V592 and the *VdΔaro2* mutant, using thresholds of |log_2_ (fold change)| ≥ 1.5 and FDR < 0.05. Among these, 2404 genes were up-regulated and 2276 were down-regulated in the mutant (Figure 5C and Supplementary Table S3). Gene Ontology (GO) classification of these DEGs revealed that the most significantly enriched biological processes included “cellular process” (1375 genes), “metabolic process” (1333 genes), and “biological regulation” (390 genes) (Figure 5D). Given the primary association of *VdARO2* with cellular and metabolic functions, DEGs from these two categories were selected for KEGG pathway enrichment analysis. The results indicated that the *VdΔaro2* mutation significantly affected pathways including “Ribosome”, “Biosynthesis of amino acids”, “Phenylalanine, tyrosine and tryptophan biosynthesis”, and “Starch and sucrose metabolism” (Figure 5E). These findings suggest that the loss of *VdARO2* not only directly disrupts the biosynthesis of aromatic amino acids but also induces a broader metabolic crisis.

### 3.6. VdARO2 Knockout Triggers a Feedback Loop in the Shikimate Pathway and Activates the Cross-Pathway Control Regulator VdCPC1

**Figure 6:**
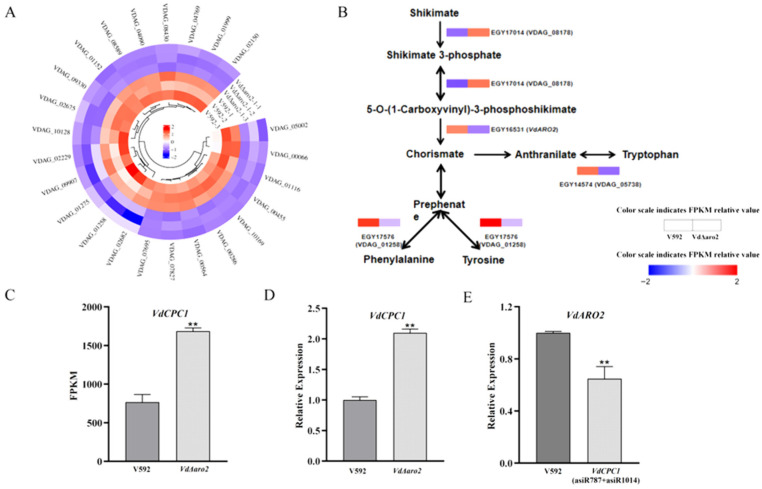
Transcriptomic Profiling Reveals Severe Disruption of Amino Acid and Central Carbon Metabolism in *VdΔaro2*. (**A**) Expression patterns of differentially expressed genes (DEGs) involved in amino acid biosynthesis. (**B**) Transcriptional changes in key genes in the phenylalanine, tyrosine, and tryptophan biosynthesis pathway. (**C**) RT-qPCR validation of *VdCPC1* up-regulation in the *VdΔaro2* mutant. ** *p* < 0.01 versus the respective control. (**D**) RT-qPCR analysis showed that the expression of *VdCPC1* was up-regulated in *VdARO2* knockout strains. Data are presented as mean ± SD (*n* = 3); ** *p* < 0.01 versus the respective control. (**E**) RT-qPCR analysis showed that the expression of *VdARO2* was down-regulated in *VdCPC1*-silenced strains. Data are presented as mean ± SD (*n* = 3); ** *p* < 0.01 versus the respective control. The darker color is the expression level of V592 wild-type strain, and the lighter color is the expression level of knockout or asiRNA strain.

KEGG analysis identified significant enrichment in amino acid biosynthesis pathways, particularly the phenylalanine, tyrosine, and tryptophan biosynthesis pathway. Given that *VdARO2* encodes a crucial enzyme for aromatic amino acid synthesis, we focused on these pathways. Transcriptome data showed that 24 genes involved in amino acid biosynthesis were significantly down-regulated in the *VdΔaro2* mutant (Figure 6A). Within the aromatic amino acid pathway, the upstream gene *ARO1* (VDAG_08178) was up-regulated, potentially as a compensatory feedback response to the blocked chorismate synthesis, while the downstream gene *GOT2* (VDAG_01258), involved in tyrosine/phenylalanine synthesis, was down-regulated (Figure 6B). This expression pattern is consistent with an interrupted aromatic amino acid pathway. Furthermore, akin to the role of *VlcpcA* in *V. longisporum*, we found that the expression of the cross-pathway control regulator *VdCPC1* was significantly increased in the *VdΔaro2* mutant, as confirmed by both transcriptomic data and RT-qPCR (Figure 6C,D). Conversely, *VdARO2* expression was reduced in *VdCPC1*-silenced strains (Figure 6E). In summary, knockout of *VdARO2* disrupts amino acid synthesis. The induction of *VdCPC1* under these conditions suggests that the cross-pathway control system is activated in response to amino acid limitation, which may be critical for *V. dahliae* to adapt to the nutrient-scarce xylem environment during host colonization.

### 3.7. The Deficiency of Microsclerotia in VdΔaro2 Is Linked to Downregulation of Key Morphogenetic and Melanin Biosynthesis Genes

**Figure 7:**
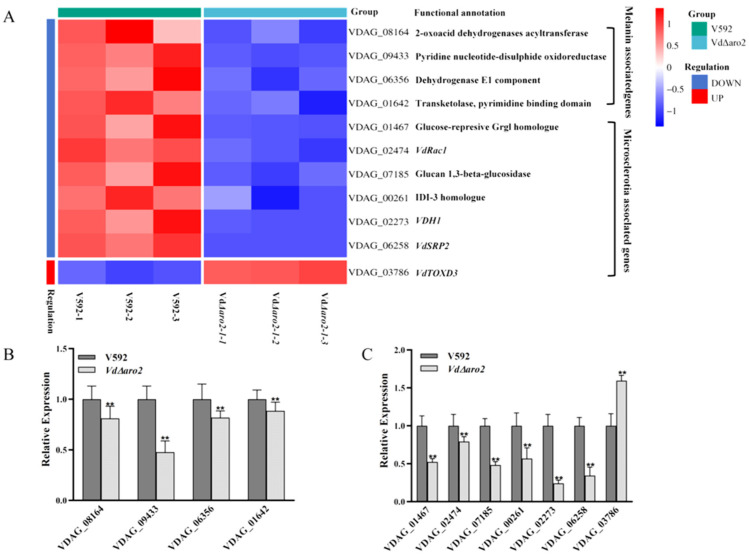
*VdARO2* knockout alters the transcription of genes governing microsclerotia formation and melanin biosynthesis. (**A**) Heatmaps showing expression patterns of key genes involved in microsclerotia development (**left**) and melanin biosynthesis (**right**). (**B**,**C**) RT-qPCR validation of selected differentially expressed genes related to (**B**) melanin synthesis and (**C**) microsclerotia formation. Data are presented as mean ± SD (*n* = 3); ** *p* < 0.01 versus the wild-type V592.

Microsclerotia, which are dormant structures formed by the melanization of mycelial aggregates, are critical for the stress resistance and disease cycle of *V. dahliae*. Functional genomic studies have identified several genes essential for microsclerotia production, including the small GTPase *VdRac1*, the hydrophobin *VDH1*, and others. Our RNA-seq analysis revealed that these key microsclerotia-related genes were significantly down-regulated in the *VdΔaro2* mutant. Melanin accumulation, a hallmark of microsclerotial maturation, depends on the precursor acetyl-CoA. We found that four genes involved in acetyl-CoA metabolism were also down-regulated in the mutant (Figure 7A). The differential expression of these critical genes was confirmed by qRT-PCR (Figure 7B,C). The expression results detected by RT-qPCR were highly consistent with the results of RNA-seq analysis, which further confirmed the reliability of the transcriptome data we obtained. In summary, integrating phenotypic observations with transcriptomic data, we demonstrate that *VdARO2* is required for the regulation of microsclerotia and melanin production in *V. dahliae*.

### 3.8. VdARO2 Deficiency Impairs Carbon Source Utilization and Energy Metabolism

To investigate the molecular mechanism by which *VdARO2* gene deletion affects carbon metabolism in *V. dahliae*, KEGG pathway enrichment analysis was performed, revealing significant enrichment of differentially expressed genes in carbon metabolic pathways. Further analysis identified 20 key carbon metabolism genes that were down-regulated in the *VdΔaro2* mutant, including 6 glycolytic pathway genes (*IDNK*, *PGLS*, etc.), 4 pentose phosphate pathway genes (*TALDO1*, *RPIB*, etc.), and 12 glyoxylate cycle pathway genes (*PDHA*, *PDHB*, etc.) (Supplementary Figure S4). Concurrently, 10 hydrolase genes involved in starch and sucrose metabolism, including *OTSA*, *TREH*, and *AMY*, also showed significant down-regulation (Supplementary Figure S5). These results demonstrate that the loss of *VdARO2* leads to coordinated down-regulation of genes involved in carbon catabolism, impairing energy production and biosynthetic precursor supply. In conclusion, our study reveals that *VdARO2* regulates gene expression in the carbon metabolic network, affecting nutrient acquisition in *V. dahliae*, which likely constitutes the metabolic basis for the observed slow growth and reduced sporulation in the *VdΔaro2* mutant.

### 3.9. VdARO2 Is Involved in the Remodeling of Glycerophospholipid Metabolism and Affects the Adaptability of Membrane Stress

To determine whether *VdARO2* deletion affects membrane composition, we analyzed the transcriptomic data and identified significant up-regulation of four glycerophospholipid metabolism genes (*TGL4*, *PCYT2*, *PLDL2*, and *GDE1*) in the *VdΔaro2* mutant (Supplementary Figure S6). Given that glycerophospholipids are fundamental structural components of cellular membranes, we hypothesize that the altered expression of these genes may lead to modifications in membrane architecture and properties. These modifications could, in turn, enhance cellular stress resistance while simultaneously impairing pathogenic functions. We therefore conclude that *VdARO2*-mediated regulation of glycerophospholipid metabolism may contribute to the observed phenotypes of enhanced stress tolerance and reduced virulence in the mutant.

## 4. Discussion

The findings of this study are consistent with observations in other pathogenic fungi that disruption of basal metabolic genes impairs growth and virulence. However, our work goes beyond this general recognition. For the first time, we elucidated in *V. dahliae* that a functional regulatory axis is formed between the aromatic amino acid biosynthesis gene *VdARO2* and the cross-pathway regulatory core factor *VdCPC1*. We confirmed that there is a two-way transcriptional regulation relationship between the two: the knockout of *VdARO2* triggers a significant up-regulation of *VdCPC1* (Figure 6C,D), while the silencing of *VdCPC1* leads to a decrease in the expression of *VdARO2* (Figure 6E). This interdependent regulatory circuit represents a sophisticated adaptive strategy that enables fungi to integrate internal metabolic states with external stress responses.

The biosynthesis of aromatic amino acids (phenylalanine, tyrosine, and tryptophan) via the shikimate pathway plays an indispensable role in the survival, pathogenicity, and environmental adaptation of *V. dahliae*. Beyond serving as essential building blocks for protein synthesis, this pathway functions as a central metabolic hub whose products are directly linked to key virulence factors required for successful host infection and the development of *Verticillium* wilt [55,56,57]. The ability to synthesize aromatic amino acids autonomously is particularly critical for *V. dahliae*, which must proliferate within the nutrient-limited and host-defensive environment of the plant xylem. Moreover, intermediates and end products of this pathway serve as precursors for secondary metabolites such as melanin and toxins [58,59]. Melanin, in particular, is essential for the formation and stress resistance of microsclerotia—dormant structures vital for the long-term survival and disease cycle of *V. dahliae*. Thus, the shikimate pathway not only supports basic cellular functions but also underlies the pathogenicity of this fungus, making its key enzymes promising targets for novel antifungal strategies.

The Vd*Δaro2* mutant exhibited a complete loss of microsclerotia formation—a phenotype consistent with studies in other fungi where disruptions in amino acid biosynthesis lead to developmental abnormalities [16,20]. Transcriptomic analysis revealed downregulation of key microsclerotia-related genes (e.g., *VdRac1*, *VDH1*) and melanin biosynthesis genes, supporting the observed defect. Since microsclerotia formation is often induced under nutrient stress, we propose that the chronic intracellular amino acid starvation caused by *VdARO2* deletion disrupts the normal stress-signaling network, preventing the transition from vegetative growth to dormancy. This metabolic impairment also led to downregulation of genes involved in carbon and energy metabolism, contributing to reduced growth and sporulation [60,61,62,63]. These results highlight how a primary metabolic defect can trigger systemic failure in fungal development and energy metabolism.

We further established the central role of *VdCPC1* in the amino acid starvation response. The hypersensitivity of *VdCPC1*-silenced strains to 5-methyltryptophan (5-MT) aligns with phenotypes observed in CPC mutants of *Neurospora crassa* and *Aspergillus nidulans* [64,65], underscoring the evolutionary conservation of the cross-pathway control system in filamentous fungi. Notably, *VdCPC1* was significantly upregulated in the Vd*Δaro2* mutant, indicating that disruption of aromatic amino acid biosynthesis activates this compensatory regulatory pathway. This result is consistent with reports in *Verticillium longisporum* [4] and extends them to *V. dahliae*. We hypothesize that while wild-type *V. dahliae* activates *VdCPC1* in response to external nutrient limitation in the xylem, the Vd*Δaro2* mutant experiences constitutive internal starvation. Despite strong induction of *VdCPC1*, the absence of a functional biosynthetic enzyme renders the compensatory response ineffective, leading to severe virulence attenuation.

Transcriptome profiling indicated that the impact of *VdARO2* deletion extends beyond aromatic amino acid metabolism. Widespread downregulation of carbon metabolism genes suggests a systemic energy crisis [66,67]. These metabolic disruptions collectively undermine pathogenic growth in planta.

Additionally, upregulation of glycerophospholipid metabolism genes (*TGL4*, *PCYT2*, etc.) may reflect membrane remodeling in response to metabolic stress—a phenomenon documented in yeast and mammalian cells [68,69,70]. Although such changes may enhance membrane stability, they could also impair fluidity and secretory efficiency, potentially affecting the delivery of effectors or cell wall-degrading enzymes [71]. This trade-off between stress adaptation and virulence represents a promising area for future investigation.

In summary, this study illustrates that pathogenicity in *V. dahliae* depends on a finely tuned balance between amino acid biosynthesis and stress-responsive regulation. *VdARO2* supplies essential metabolic precursors, while *VdCPC1* modulates gene expression under nutrient limitation. Disruption of either component impairs fungal development and virulence. These insights open avenues for novel control strategies, such as dual-target HIGS against *VdARO2* and *VdCPC1*, or small-molecule inhibitors that disrupt the cross-pathway control system. By targeting the pathogen’s adaptive metabolic network, such approaches may offer effective and sustainable management of *Verticillium* wilt.

## 5. Conclusions

This study systematically elucidates the key metabolic regulatory mechanisms governing the pathogenicity of *V. dahliae*, revealing the central role of a sophisticated regulatory network formed by the aromatic amino acid biosynthetic gene *VdARO2* and the amino acid starvation response regulator *VdCPC1* during host colonization. Functional impairment of *VdARO2*, which encodes a key enzyme in aromatic amino acid biosynthesis, not only severely restricts vegetative growth and conidiation but also completely abolishes microsclerotia formation, while simultaneously triggering global metabolic dysregulation—including disruption of central carbon metabolism. This metabolic stress markedly upregulates *VdCPC1* expression, activating its mediated stress response pathway. Further mechanistic investigation demonstrates synergistic action between these genes during infection: *VdARO2* provides essential metabolic support for fungal development and pathogenic structure formation, whereas *VdCPC1* coordinates global gene expression and remodels metabolic networks to enhance adaptability in response to host-imposed stresses. Disruption of either component within this regulatory network severely compromises fungal virulence. These findings not only advance our understanding of the molecular pathogenesis of plant-pathogenic fungi, but also establish a solid theoretical foundation and identify promising molecular targets for novel control strategies based on interference with amino acid metabolic pathways.

## Data Availability

The original contributions presented in this study are included in the article/Supplementary Materials. Further inquiries can be directed to the corresponding author.

## Supplementary Materials

The following supporting information can be downloaded at: https://www.mdpi.com/article/10.3390/microorganisms13122852/s1, Figure S1: The *VdARO2* gene knockout module diagram, *VdARO2* gene knockout, *VdCPC1* asiRNA-treated and HIGS (*pTRV2-VdCPC1*) the verification of the mutant. Figure S2: Time-course of host-induced *VdARO2* expression during cotton root co-culture. Figure S3: The qPCR melting curve was used to show the data screening criteria. Figure S4: *VdARO2* knockout disrupts carbon metabolism pathways in *V. dahliae.* Figure S5: *VdARO2* deficiency leads to downregulation of starch and sucrose metabolism genes. Figure S6: *VdARO2* knockout induces remodeling of glycerophospholipid metabolism. Table S1: Oligonucleotides and asiRNAs used in this study; Table S2: Quality control data statistics table. Table S3: List of significantly differentially expressed genes (DEGs) in the *VdΔaro2* mutant vs. wild-type.
