# Supplementary material for: The Aromatic Amino Acid Biosynthesis Gene VdARO2 and the Cross-Pathway Regulator VdCPC1 Coordinately Regulate Virulence in Verticillium dahliae
Source: Microorganisms. 2025 Dec 15;13(12):2852. doi: 10.3390/microorganisms13122852 (PMC12736186; doi:10.3390/microorganisms13122852)
Supplement: Supplementary file 1 [file microorganisms-13-02852-s001.zip › Supplementary Figure.pdf]

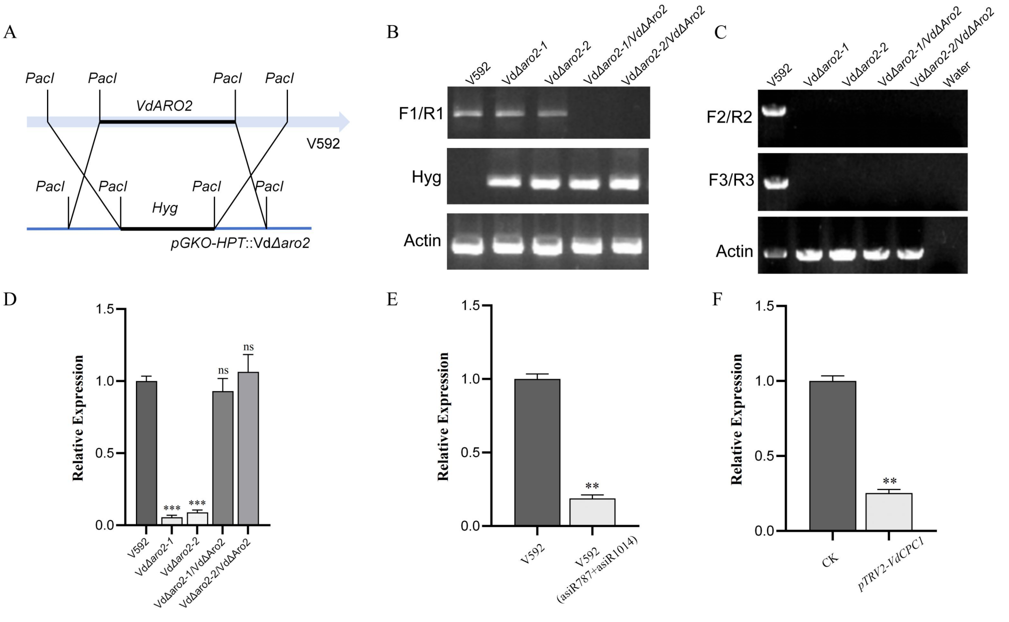

Figure S1. **The *VdARO2* gene knockout module diagram, *VdARO2* gene knockout, *VdCPC1* asiRNA-treated and HIGS silencing (*pTRV2-VdCPC1*) the verification of the mutant. (A) Homologous recombination targeted destruction of *VdARO2* strategy. The wild-type *VdARO2* locus with a flanking region was replaced with a hygromycin B resistance cassette (Hyg). (B) PCR detection of *VdARO2* gene in knockout transformants. The knockout mutants were screened with primers *VdARO2*-F1/R1, respectively. *β-tubulin* was used as an internal reference. Swimming lane 1 represented the gene expression in the wild-type strain, swimming lane 2,3 represented the gene expression in the complementary mutant strain, and swimming lane 4,5 represented the gene expression in the knockout mutant strain. (C) The knockout and complementary mutants were detected by ectopic insertion PCR using *VdARO2*-F2/R2 and *VdARO2*-F3/R3 primers. *β-tubulin* was used as an internal reference. lane 1 represents the gene expression of the wild-type strain, lanes 2,3 represent the gene expression of the complementary mutant strain, lanes 4,5 represent the gene expression of the knockout mutant strain, lane 6 is the shadow control. *VdARO2*-F2/R2 is upstream homologous arm to gene interior, and *VdARO2*-F3/R3 is gene interior to downstream homologous arm. (D) The abundance of *VdARO2* gene transcripts in *VdARO2* knockout mutant lines was verified by qRT-PCR. (E) The transcript abundance of *VdCPC1* gene in *VdCPC1* asiRNA-treated mutant lines was verified by qRT-PCR. (F) The transcript abundance of *VdCPC1* gene in HIGS silencing (*pTRV2-VdCPC1*) was verified by qRT-PCR. *p < 0.05; **p < 0.01; ***p < 0.001 (Student's *t*-test).**

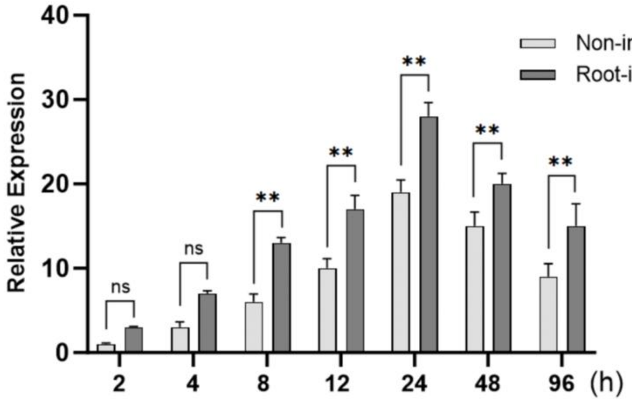

Figure S2. **Time-course of host-induced *VdARO2* expression during cotton root co-culture. Fold-change relative to non-induced control. Data are mean ± SD (n = 3). Significant induction at 24 hpi (*** p < 0.001, Student's *t*-test).**

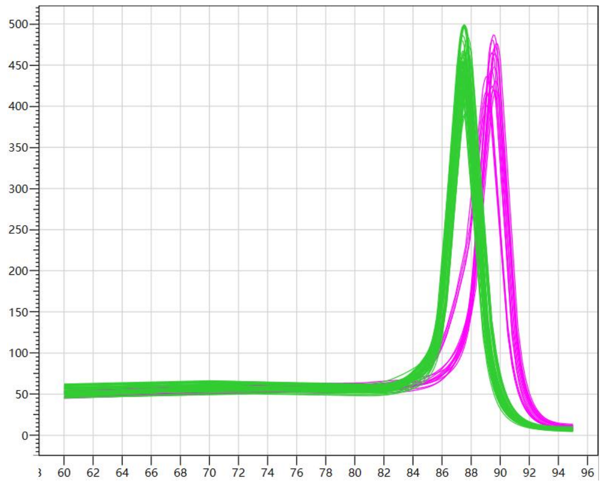

Figure S3. The qPCR melting curve was used to show the data screening criteria. Curves with a single, sharp peak were considered specific amplification and included in the final analysis. The stringent filtering based on melt curve profile ensured the reliability of the gene expression data presented in this study.

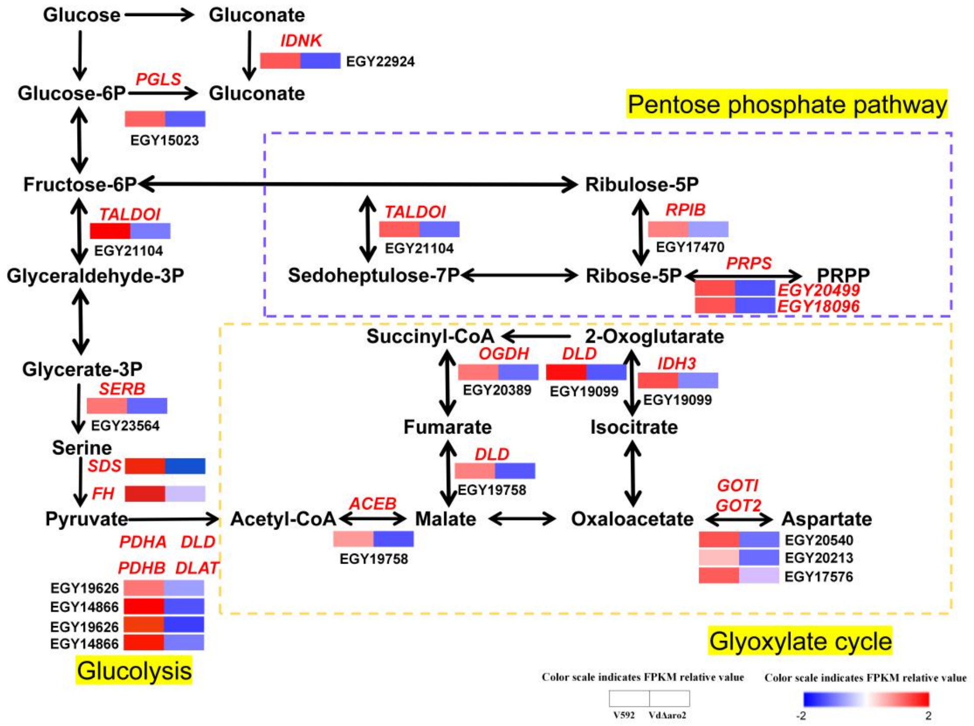

Figure S4. *VdARO2* knockout disrupts carbon metabolism pathways in *V. dahliae.* including glycolysis, pentose phosphate pathway, and glyoxylate cycle, showing the expression changes of associated genes in the *VdΔaro2* mutant compared to the wild-type V592. Genes highlighted in blue indicate downregulation in the mutant. Key enzymes such as *IDNK*, *PGLS*, *TALDO1*, *RPIB*, *PDHA*, *PDHB*, and others involved in carbon utilization are significantly suppressed.

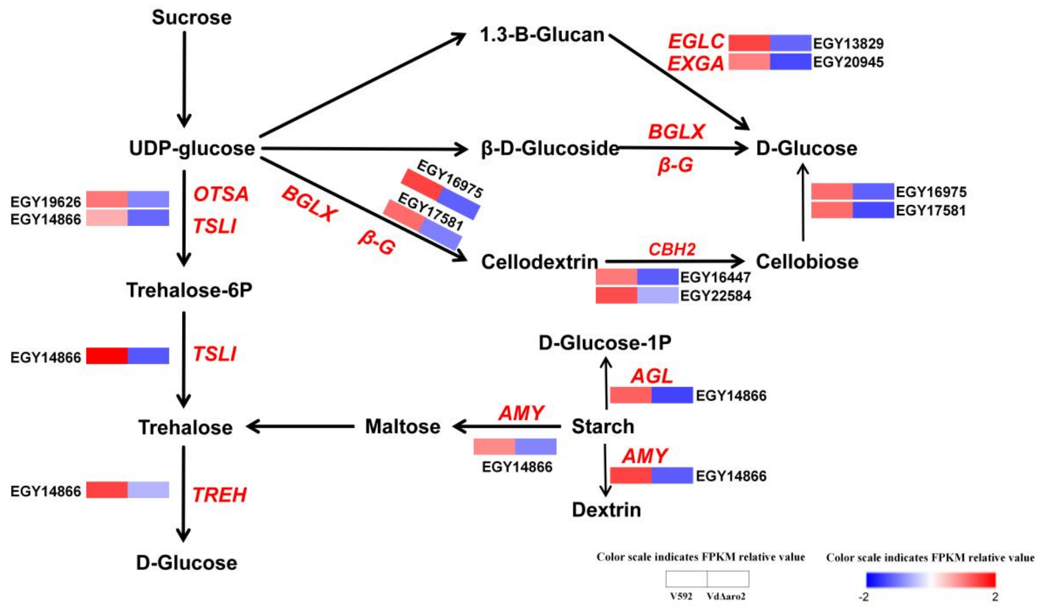

Figure S5. *VdARO2* deficiency leads to downregulation of starch and sucrose metabolism genes. Genes such as *TREH*, *AMY*, *BGLX*, and *EGLC* are significantly downregulated in the *VdΔaro2* mutant, suggesting a reduced capacity to utilize complex carbon sources, which may contribute to the observed growth and sporulation defects.

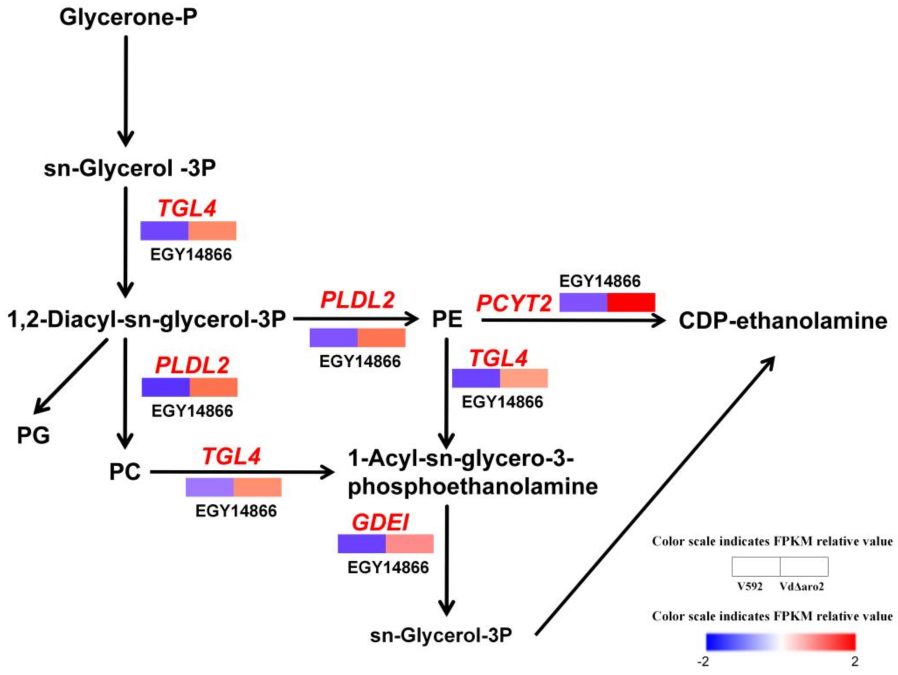

Figure S6. *VdARO2* knockout induces remodeling of glycerophospholipid metabolism. Expression profile of genes involved in glycerophospholipid metabolism in the *VdΔaro2* mutant, Key genes such as *TGL4* (triacylglycerol lipase), *PCYT2* (choline-phosphate cytidylyltransferase), *PLDL2* (phospholipase D-like 2), and *GDE1* (glycerophosphodiester phosphodiesterase) are upregulated.
